# Hydrological and biogeochemical response of the Mediterranean Sea to freshwater flow changes for the end of the 21^st^ century

**DOI:** 10.1371/journal.pone.0192174

**Published:** 2018-02-15

**Authors:** Diego Macias, Adolf Stips, Elisa Garcia-Gorriz, Alessandro Dosio

**Affiliations:** European Commission, Joint Research Centre, Ispra, Varese, Italy; University of Vigo, SPAIN

## Abstract

We evaluate the changes on the hydrological (temperature and salinity) and biogeochemical (phytoplankton biomass) characteristics of the Mediterranean Sea induced by freshwater flow modifications under two different scenarios for the end of the 21^st^ century. An ensemble of four regional climate model realizations using different global circulation models at the boundary and different emission scenarios are used to force a single ocean model for the Mediterranean Sea. Freshwater flow is modified according to the simulated changes in the precipitation rates for the different rivers’ catchment regions. To isolate the effect resulting from a change in freshwater flow, model results are evaluated against a ‘baseline’ simulation realized assuming a constant inflow equivalent to climatologic values. Our model results indicate that sea surface salinity could be significantly altered by freshwater flow modification in specific regions and that the affected area and the sign of the anomaly are highly dependent on the used climate model and emission scenario. Sea surface temperature and phytoplankton biomass, on the contrary, show no coherent spatial pattern but a rather widespread scattered response. We found in open-water regions a significant negative relationship between sea surface temperature anomalies and phytoplankton biomass anomalies. This indicates that freshwater flow modification could alter the vertical stability of the water column throughout the Mediterranean Sea, by changing the strength of vertical mixing and consequently upper water fertilization. In coastal regions, however, the correlation between sea temperature anomalies and phytoplankton biomass is positive, indicating a larger importance of the physiological control of growth rates by temperature.

## Introduction

The Mediterranean Sea has been described as a hot-spot for climate change [1] as it is located in a temperate region which is expected to become warmer and drier in the nearby future [2] and it hosts a very large human population that exerts a considerable influence on this marine ecosystems’ conditions [3].

The combined effect of natural and anthropogenic forcings will potentially create a wide range of pressures for the Mediterranean Sea ecosystems. Such pressures could undermine its environmental status putting at risk the ecosystems’ services provided by this European regional sea. Thus, being able to anticipate potential changes and having the opportunity to propose measures to avoid deleterious effects is a fundamental tool for EU regulations and, more specific, for the Marine Strategy Framework Directive (MSFD) [4] in order to guide a proper sustainable Blue Growth in which all marine and maritime sectors can contribute to welfare, innovation and growth.

One of the tools used to look at potential changes in future conditions in this region is the Regional Climate System model [5]. Numerical models have been mostly used to study changes in physical properties of the Mediterranean basin under different future scenarios [6–9], while assessments of potential changes on Mediterranean biological production characteristics in future scenarios are much less common [10–12].

All these previous works considered the combined effect of expected future changes in atmospheric forcing, boundary conditions and land-sea interactions (freshwater quantity and quality). However, integrated modelling systems could also provide a quantitative assessment of the relative importance of each changing element for different scenarios. For example [13] performed a series of scenario simulations for the Mediterranean Sea under different climatic scenarios but assuming constant freshwater inputs and nutrient levels to assess the effect of a changing atmospheric forcing on the hydrodynamic and biogeochemical conditions of the basin.

With the present contribution we expand this previous work by evaluating the effects on the Mediterranean basin of a change in the quantity of the freshwater flow associated to expected alterations of the precipitation rate in the different rivers’ catchment areas. The same ocean model that has already shown to provide a reasonable representation of past and present hydrodynamic and biogeochemical conditions of the Mediterranean basin [3, 14] is used in conjunction with a regional climate model (RCM) forced at the boundaries by different global climate models (GCMs) from the Coupled Model Intercomparison Project Phase 5 (CMIP5). Two different emission scenarios are evaluated, RCP4.5 and RCP8.5 [2].

Henceforth, the main aim of the present contribution is to quantitatively evaluate the effects of a change in the freshwater flow for the Mediterranean Sea ecosystem under different climate scenarios. Changes in the hydrodynamic (e.g., thermo-haline properties) and biogeochemical (e.g., chlorophyll) conditions will be evaluated by comparing the ecosystems’ conditions simulated for the future assuming no change in freshwater flow and those simulated imposing alterations of freshwater inputs from rivers.

## Materials and methods

### Ocean model

We applied the 3-D General Estuarine Transport Model (GETM) simulate the hydrodynamics in the Mediterranean Sea. GETM solves the three-dimensional hydrostatic equations of motion applying the Boussinesq approximation and the eddy viscosity assumption [15] as detailed in [16] and at http://www.getm.eu.

**Fig 1:**
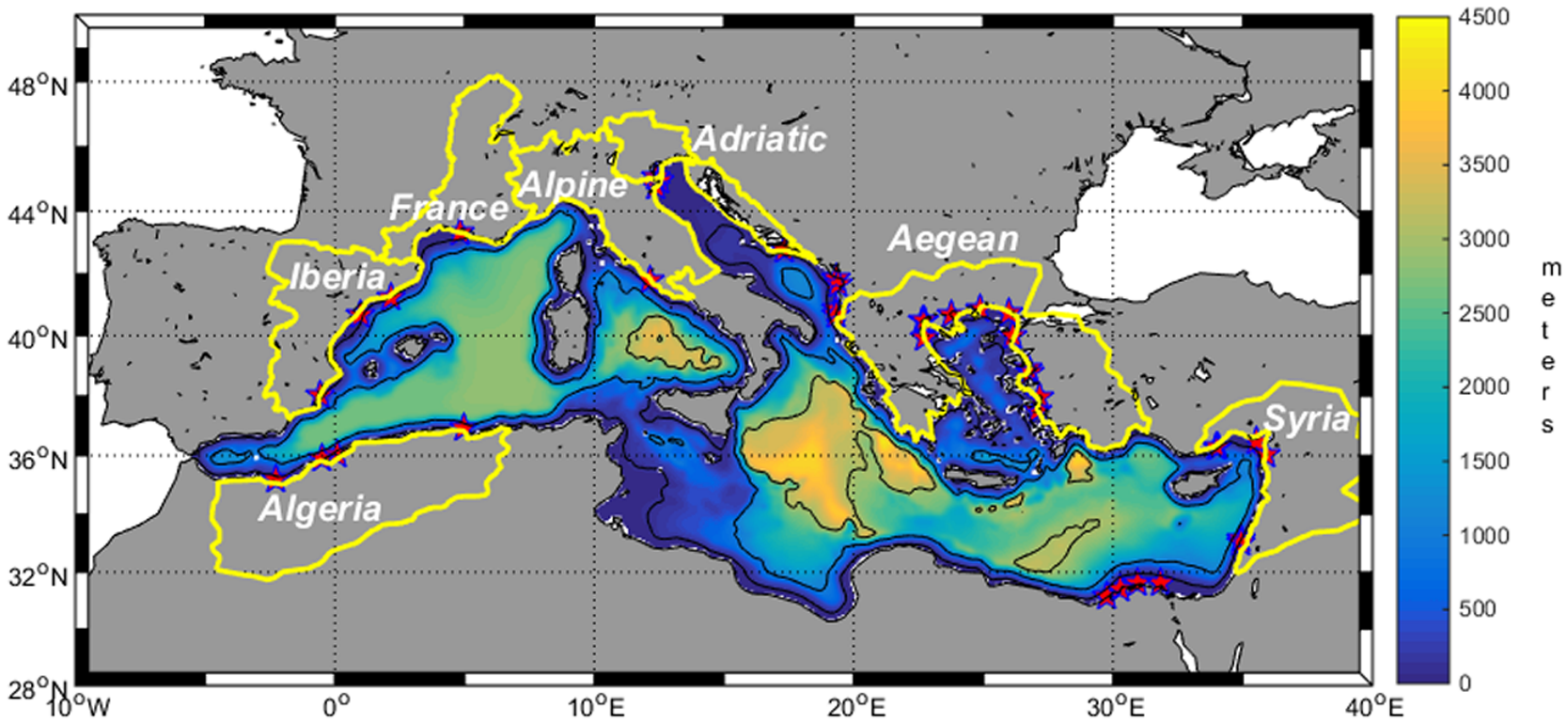
Model domain, bathymetry (background scale), included rivers (red stars) and polygons (yellow lines) defining the different regions considered for rivers’ basins.

The configuration of the Mediterranean Sea (Fig 1) presents a horizontal resolution of 5’ x 5’ and 25 vertical sigma-layers following the bottom topography. Vertical resolution of the layers is higher at the surface (where biogeochemical processes typically happen) and decreases towards the bottom. A third-order Total Variation Diminishing (TVD) advection scheme is used as recommended by [17].

The ETOPO1 (http://www.ngdc.noaa.gov/mgg/global/) database was used to build the bathymetric grid by averaging depth levels to the corresponding horizontal resolution of the model grid. The salinity and temperature climatologies used as initial conditions at the start of the model integration were obtained from the Mediterranean Data Archeology and Rescue-MEDAR/MEDATLAS database (http://www.ifremer.fr/medar/) while biogeochemical initial and boundary conditions were computed from the World Ocean Atlas database (www.nodc.noaa.gov/OC5/indprod.html). All model runs described below started with exactly the same initial conditions.

Boundary conditions at the western entrance of the Strait of Gibraltar were also computed from the same MEDAR/MEDATLAS dataset imposing monthly climatological vertically-explicit values of salinity. Sea surface temperature at the western entrance of the Strait were extracted from the nearest node of the driven GCM for each simulation (see below) while the rest of the water column temperature was not changed. No horizontal currents were imposed at the open boundary. With this boundary configuration the circulation through the Strait is established by the internally-adjusted baroclinic balance provoked mainly by the deep-water formation within the basin [18].

The present configuration of the ocean model includes 37 rivers discharging along the Mediterranean coast (red stars in Fig 1). River inflow is treated as a boundary condition regarding water flow, temperature, salinity and nutrients (computed from the databases mentioned below) with respect to the grid cells where the river is entering. Once in the oceanic domain, the freshwater plume is subjected to the general hydrodynamic processes governing the water movements.

The GETM configuration for the Mediterranean Sea is forced at surface every 6 hours by the following atmospheric variables: wind velocity at 10 meters (U10 and V10), air temperature at 2 m (t2), specific humidity (sh), cloud cover (tcc) and sea level pressure (SLP) provided by the different realizations of the atmospheric model described in the next section. Bulk formulae are used to calculate the corresponding relevant heat, mass and momentum fluxes between atmosphere and ocean [14]. No tidal forcing is included in the present GETM implementation.

GETM is coupled online to the MedERGOM biogeochemical model [3, 19] by using the Framework for Aquatic Biogeochemical Models (FABM, https://sourceforge.net/projects/fabm/)[20]. MedERGOM is a modified version of the ERGOM model [21] specifically adapted to represent the conditions of the pelagic ecosystem of the Mediterranean Sea. It has proven useful to describe present [19], past [3] and future [13] biogeochemical conditions in this semi-enclosed basin.

To explore changes on vertical stratification of the water column in the different model runs the integrated value of the thermal expansion coefficient (α) and of the salinity contraction coefficient (β) where calculated. α represents how much the seawater increases its volume (hence reducing density) for each increment of temperature while β represents the seawater loss in volume (increase in density) with the increasing salinity value [22]. Henceforth, changes in the ratio α/β (AONB) could be used as proxy for changes in stratification strength as shown further down [23, 24].

### Regional climate models

The ocean model described above is forced at the surface with the outputs from the COSMO-CLM RCM (http://www.clm-community.eu/), in the framework of the EURO-CORDEX initiative (http://www.euro-cordex.net/). The RCM is forced by two GCMs, namely EC-Earth and MPI-ESM-MR included in the CMIP5 (Table 1). The RCM spatial resolution is 0.11°. For each GCM two emission scenarios as defined by IPCC are considered; RCP4.5 and RCP8.5 [25]. Hence a total of four member ensemble runs are analyzed in this work.

**Table 1:** Institutes/modelling groups providing the atmospheric model data used in the present contribution.

Modelling group	Driving model name	Emission scenarios
ECEARTH consortium	EC-EARTH	rcp 4.5 / rcp 8.5
Max-Planck-Institut für Meteorologie (Max Planck Institute for Meteorology)	MPI-ESM-MR	rcp 4.5 / rcp 8.5

As a previous step to the Mediterranean runs and as concluded in [26], we have carried out a bias-correction of the most relevant atmospheric variables: air temperature, cloud cover and wind intensity. As shown in [26], the atmospheric variables provided by the RCM realizations induce a severe underestimation of simulated SST for the present-day. The basic principle of the bias-correction technique consists in finding a transfer function that allows matching the cumulative distribution functions (CDFs) of modeled and observed data [27–29]. In our study, spatially-averaged values of the observed and model atmospheric variables over the entire Mediterranean Sea basin were used, so no spatially explicit correction was applied. A detailed description of the bias-correction technique and evaluation over the present climate is found at [26].

We choose COSMOS-CCLM as RCM because it has been found to provide reliable conditions to simulate oceanic conditions in the Mediterranean Sea after the bias-correction procedure described above [13, 26]. The evaluation of other RCMs as forcing conditions for our ocean model is, thus, left for future research.

### Rivers scenarios: Freshwater flow modifications

The present-day values for river discharges were derived from the Global River Data Center (GRDC, Germany) database while nutrient loads (nitrate and phosphate) of freshwater runoff were obtained from [30]. We, then, compute the seasonal climatological values of water flow and nutrient concentration for each river for the period 1985–2000. Using this climatological rivers conditions, four full-time scenario simulations covering the period 2013–2100 were performed using the four RCM realizations described above (MPI & EcEarth under RCPs 4.5 and 8.5). These continuous (2013–2100) model runs provide the ‘*baseline*’ scenario conditions for the end of the century, as they only consider the impacts of atmospheric forcing changes [13].

To estimate potential changes in freshwater flow in the different climate scenarios a proxy was created by evaluating the relative change on precipitation over the different rivers’ basins for the end of the century in a similar way as done by [7]. First of all we defined the regions containing river catchments following the definition of the Water Information System for Europe (WISE) river basin districts (http://www.eea.europa.eu/data-and-maps/data/wise-river-basin-districts-rbds-1) but grouping together sets of rivers sharing a common basin area. This way, 8 different ‘provinces’ have been defined as indicated in Table 2 and also shown in Fig 1. Unfortunately, the catchment area for the Nile is not included within the EURO-CORDEX domain, so for this river no scenario on water flow changes could be derived. However, from previous works [7], no big alterations of the freshwater flow are to be expected for the Nile.

**Table 2:** Basin names and included rivers.

Region	Rivers included in the GETM domain
*Iberia*	Llobregat; Ebro; Jucar
*France*	Rhone
*Alpine*	Adige; Po; Tiber
*Adriatic*	Neretva; Bojana; Mati; Vijosa
*Aegean*	Meric; Nestos; Gediz; Bakircay; Buyuk; Kucuk; Strimon; Axios; Pinios
*Syria*	Yarmuk; Ceyhan; Asi; Goeksu; Nahraz
*Nile*	Nile
*Algeria*	Meulouya; Bouselam; Chelif; Tafna

**Fig 2:**
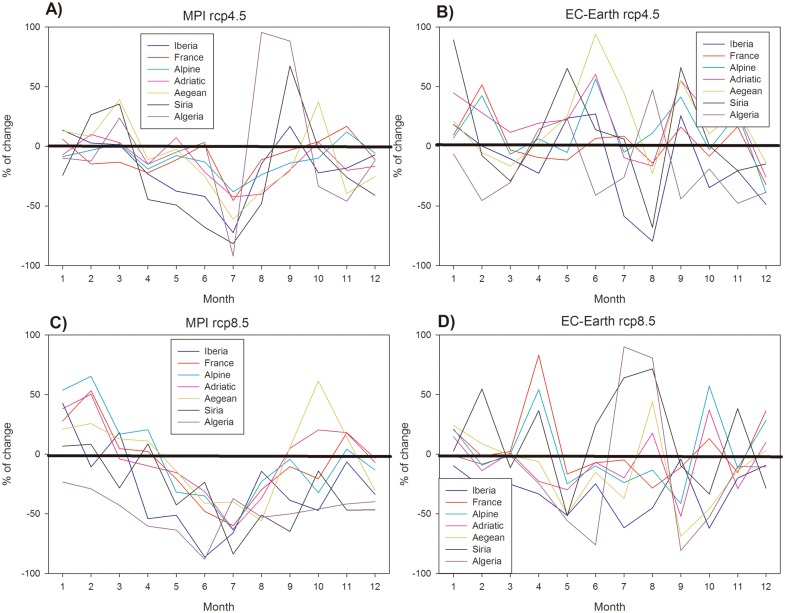
Relative change on P for each catchment area for MPI RCP4.5 scenario (A), EC-Earth RCP4.5 scenario (B), MPI RCP8.5 scenario (C) and EC-Earth RCP8.5 scenario (D). The 0% change level is indicated with a bold black line.

For the rest of the basins/rivers it is possible to compute relative changes of precipitation (P) between the first five years of the forecasting period (2014–2019) and the last five years of the simulations (2094–2099) for each combination of GCM/RCP. The monthly coefficients of relative P changes for each model/emission scenario (Fig 2) are used to generate new water flows in each river for the future conditions by multiplying these coefficients by the present-day freshwater flow values.

We then use the initial conditions corresponding to January 2090 in the *baseline* simulations [13] to start a 10 year simulation with the modified freshwater conditions for each model/scenario combination. The mean conditions from 2095–2100 were compared with the same years in the simulation with the climatologic rivers (i.e., the *baseline* simulation).

The used model configuration is, thus, exactly the same as in [13] but we add the freshwater flow modifications described above. The nutrient concentrations in the rivers are kept unchanged, as there are no solid socio-economic scenarios available for the considered time frame. From this comparison, the effect of a changing freshwater discharge on the Mediterranean Sea ecosystem could be isolated and quantitatively evaluated against, for example, the consequences of changing atmospheric conditions.

## Results

The annual average changes in P values for each model/emission scenario and for each basin are shown in Table 3. For most models/RCPs freshwater flow will decrease in the future between 9% and 0.85%, however for EcEarth under RCP4.5 the total freshwater flow will increase by about 3.2%.

**Table 3:** Computed annual changes in freshwater flow for the different model/rcp combinations.

Model / emission scenario	Freshwater flow change (% of baseline values)
MPI/rcp4.5	-326 km^3^/y (-9%)
MPI/rcp8.5	-228 km^3^/y (-6.4%)
EcEarth/rcp4.5	+114 km^3^/y (+3.2%)
EcEarth/rcp8.5	-31 km^3^/y (-0.85%)

In order to consider also the seasonality of the potential changes, the climatological P cycles for the first 5 years of the RCM realization (2014–2019) are compared to the climatological P cycles during the last 5 years of the runs (2094–2099). The percentage of change for each river’s basins are shown in Fig 2 for the different model/emission scenario combination.

**Fig 3:**
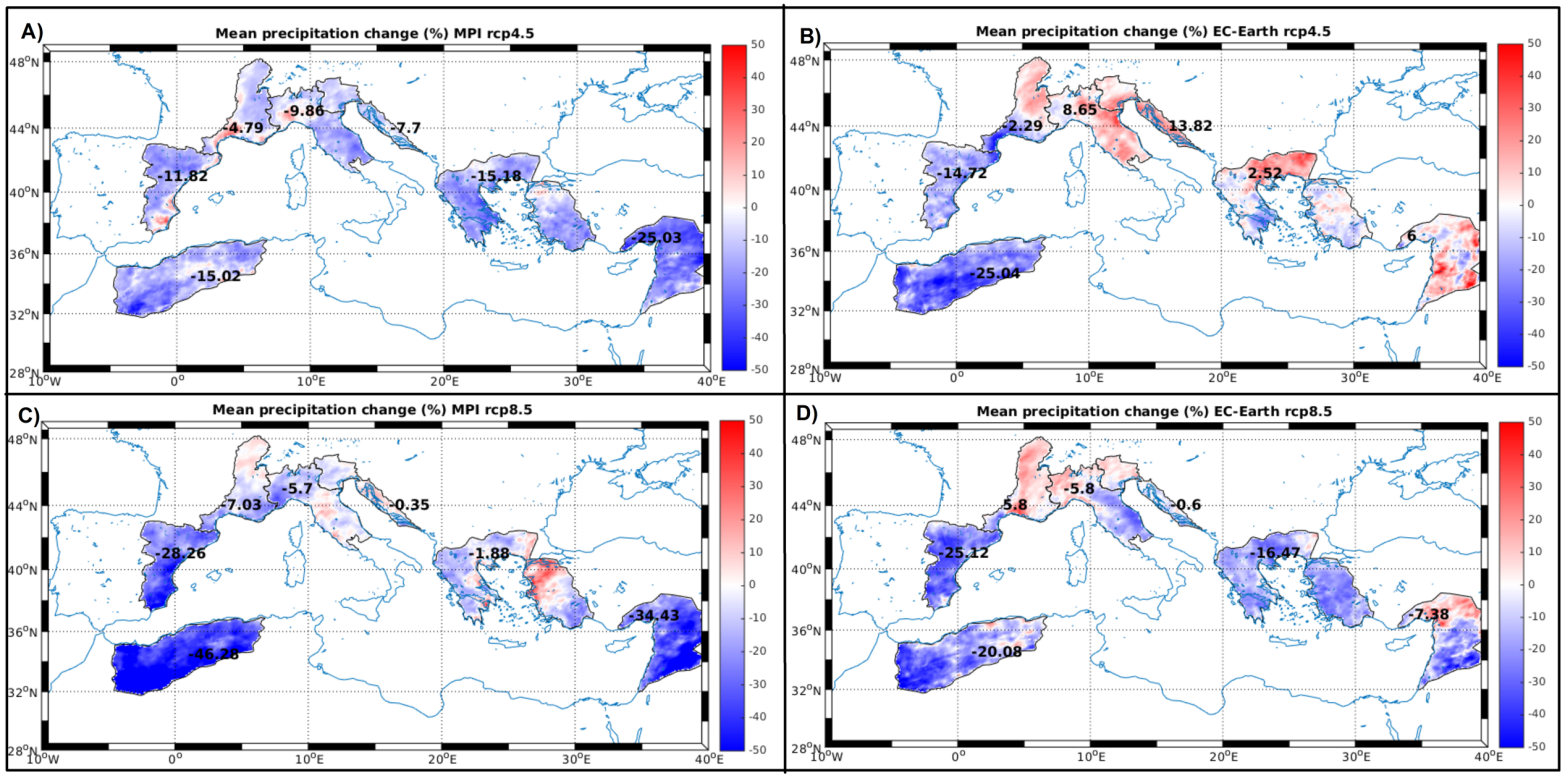
Mean P change (%) for each river basin district and model/RCP combination.

In Fig 2A and 2C, corresponding to the MPI-forced realizations, it could be seen that in most catchments future P decrease occurs mainly during summer. In winter/spring and during fall they tend to remain close to actual values or to slightly increase. The maps of mean anomalies for this GCM (Fig 3A and 3C) show a generalized reduction of P in all river basin districts with the larger decreases in the south and eastern regions. The *Adriatic* and *Aegean* provinces show the lower decreases, especially under RCP8.5.

No clear seasonal pattern could be identified on the EC-Earth forced scenarios (Fig 2B and 2C) as they show a much larger inter-catchment variability. The spatial distribution of the P anomalies for this model (Fig 3B and 3D) show a different pattern than MPI, with the northern part of the Mediterranean becoming wetter in the future, especially under RCP4.5.

The effect of changing freshwater flow in the Mediterranean ecosystems is investigated by comparing the simulations results obtained with the climatological rivers [13] with those derived from the rivers scenarios described above for the period 2095–2100.

**Fig 4:**
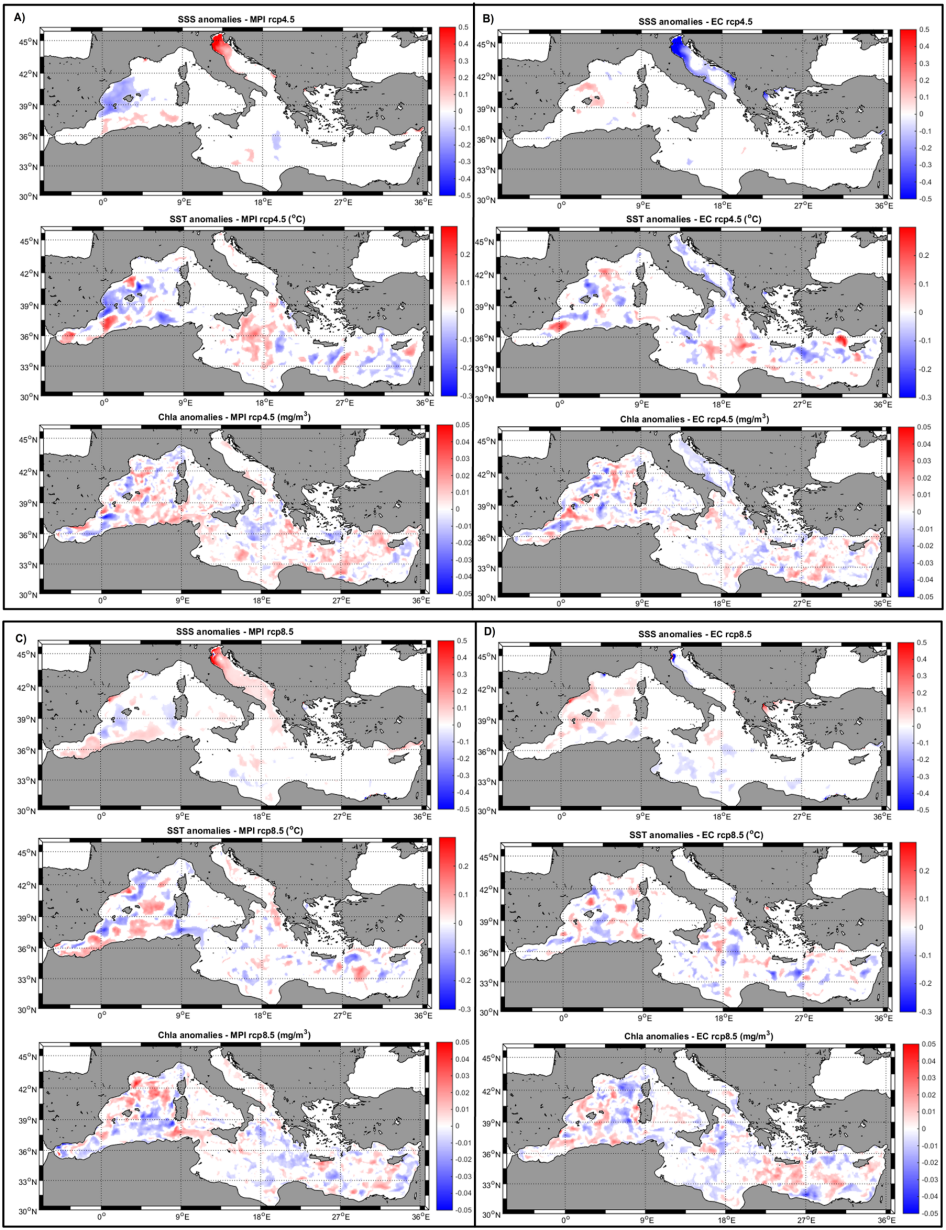
SSS, SST and Cla anomalies maps (simulation flow modified—simulation flow cte) for the period 2095–2100 and for the different models/emission scenarios.

For sea surface salinity (SSS), relative mean differences (Table 4) are around 0.05%-0.1% for the different models’ realizations. However, on the maps some spatial patterns could be identified (Fig 4). For MPI-RCP4.5 there is an increase in the salinity of the Adriatic Sea and a decrease in the Balearic Sea. For EC-RCP4.5, there is a generalized decrease of SSS in the Adriatic. Salinity anomalies for MPI-RCP8.5 are quite similar to the lower emission scenario but with a larger increase within the Adriatic while no coherent pattern could be found for EC-RCP8.5.

**Table 4:** Basin-integrated anomalies for the different model/emission scenario runs (absolute and relative changes with respect to the baseline future).

Driving model	Emission scenario	ΔSST	ΔSSS	ΔChla
MPI	rcp4.5	-3.8*10^−3^°C (-0.018%)	0.018 (0.05%)	1.2*10^−3^ mg/m^3^ (0.46%)
	rcp8.5	7.4*10^−4^°C (0.003%)	0.046 (0.11%)	5.9*10^−4^ mg/m^3^ (0.21%)
EcEarth	rcp4.5	-8.6*10^−3^°C (-0.042%)	-0.053 (-0.13%)	-7.6*10^−4^ mg/m^3^ (-0.27%)
	rcp8.5	-9.1*10^−3^°C (-0.04%)	0.015 (0.04%)	1.0*10^−3^ mg/m^3^ (0.38%)

Sea surface temperature (SST) anomalies maps (Fig 4) do not show a coherent spatial pattern but rather scattered positive/negative areas throughout the entire basin for all models/scenarios considered in this work. The mean basin-wide SST anomalies are very small for all scenarios, being always below 0.1% of relative difference (Table 4).

We can also examine the anomaly maps for the phytoplankton biomass in terms of chlorophyll (Chla) concentration (Fig 4). In general, these maps show that there is not a clear pattern in the simulated anomalies with scattered distributions of positive and negative patches throughout the entire basin. The same type of patchy, non-structured distribution could be found in the primary production rate (PPR) anomaly maps (not shown). The mean Chla value in the entire basin does not change significantly with the modification of the water flow from the rivers (Table 4).

**Fig 5:**
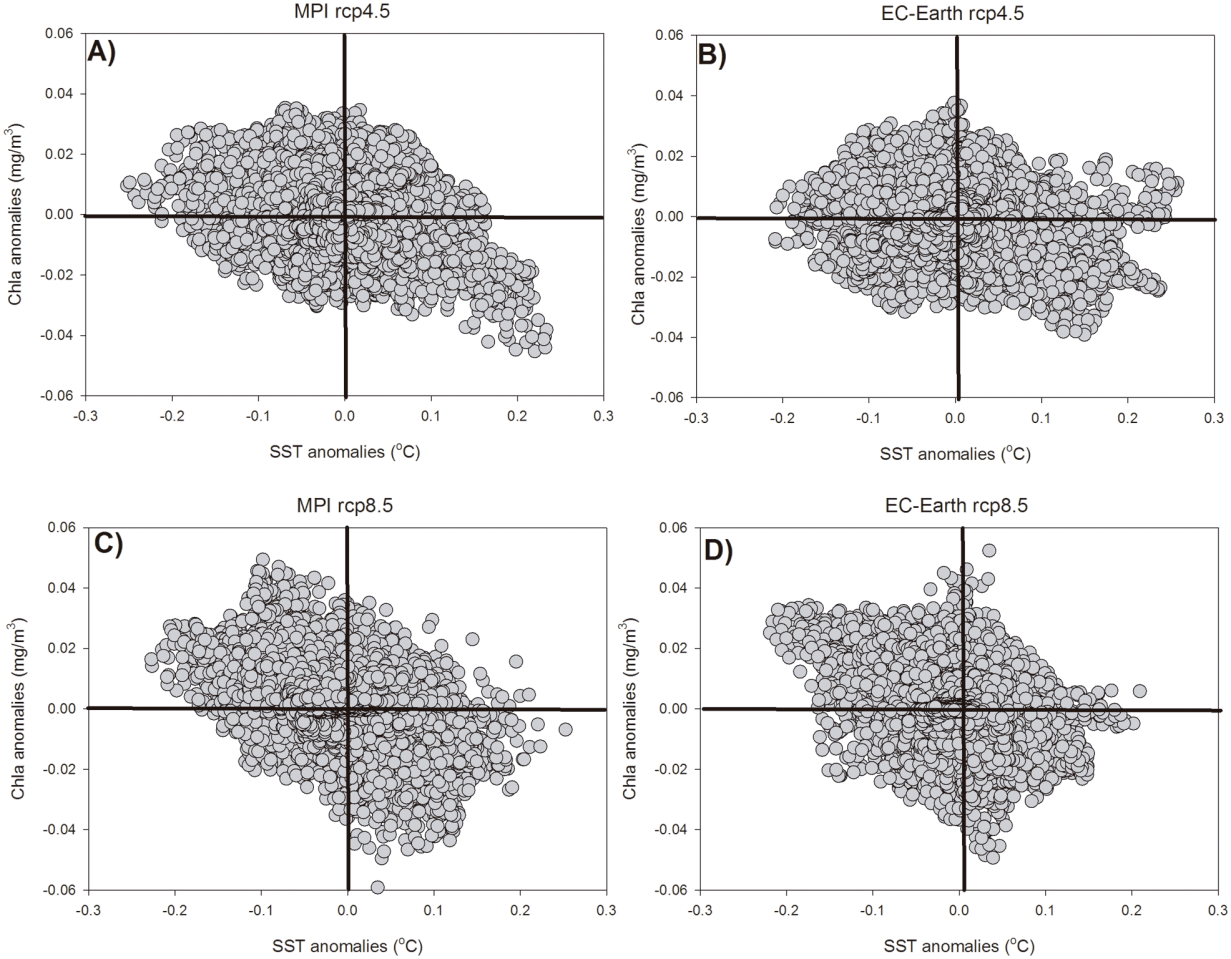
Scatter plots of SST anomalies versus Chla anomalies for the different models/emission scenarios.

It is, however, striking that the spatial distributions of SST anomalies and those of phytoplankton biomass seem to present certain similarities. Actually, if the scatter of biomass versus SST anomalies is plotted (Fig 5) a general negative relationship is suggested, with reduced biomass (and production) in regions where SST is increased *(i*.*e*., where stratification is enhanced), although a large dispersion is observed in all scatter plots. This general relationship is more evident using phytoplankton biomass anomaly than PPR (not shown) and for MPI rather than for EC-Earth simulations (Fig 5).

**Fig 6:**
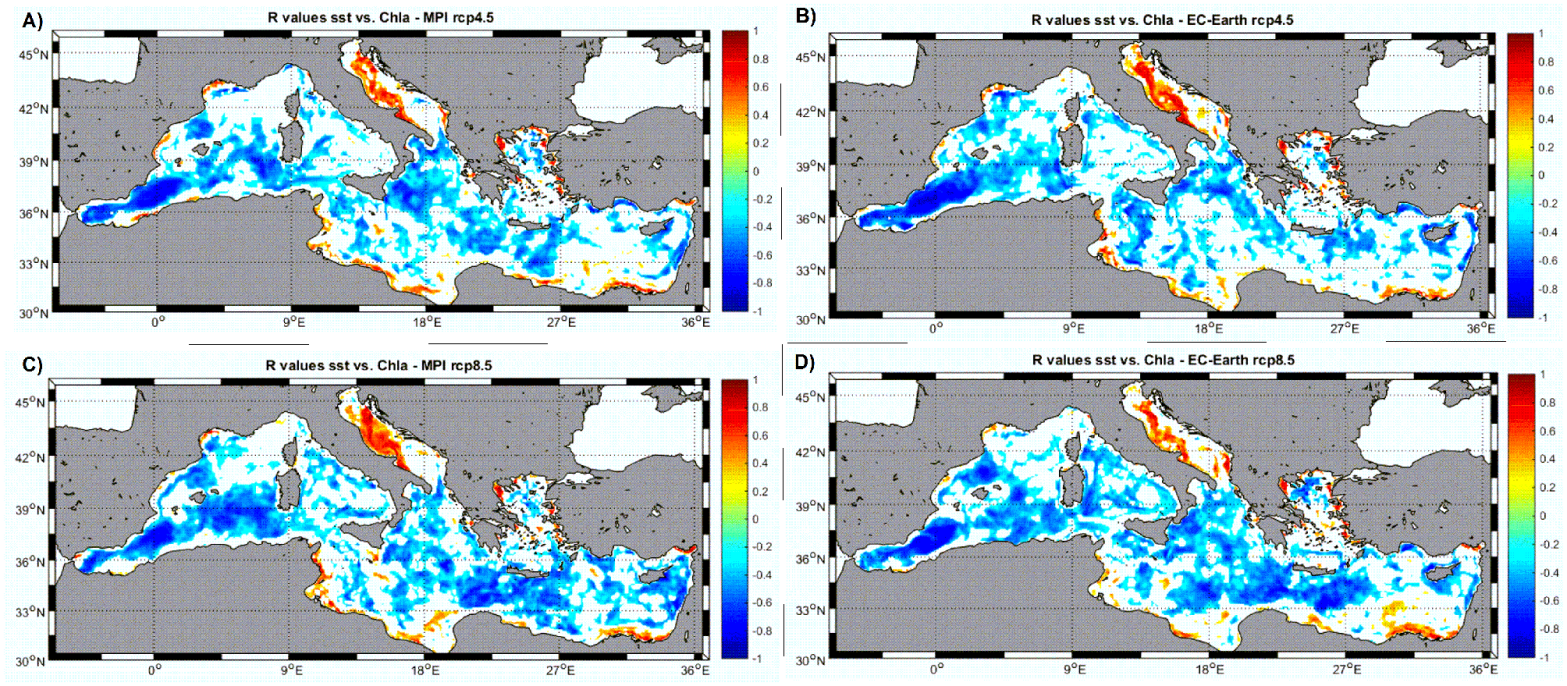
Correlation coefficient (R) maps of monthly SST anomalies vs. Chla anomalies at each model node for the different models/emission scenarios. Non-significant correlations (at 99%) are blanked.

In order to further understand the mechanisms creating the general relationships shown above, and to try to identify why dispersions in the scatters are so large, correlation maps between monthly SST anomalies and phytoplankton biomass anomalies at each model node are shown in Fig 6. In these correlation maps a common pattern seems to emerge. There is, in general, a strong negative correlation between SST anomalies and phytoplankton biomass in the open sea regions for all combinations of model/emission scenarios (blue areas in Fig 6). However, in many coastal areas and marginal basins (such as the Adriatic and Aegean Seas) the correlation is equally strong but with positive sign (red color regions in Fig 6). There, warmer waters are associated with enhanced biological activity.

The statistics of the correlation analysis (Table 5) indicate that positive correlations are found in shallower regions (average depth 283 m) and in a smaller fraction of the basin (~12% of the total sea area) while negative correlations are more widespread (covering ~ 60% of the basin) and typically found in much deeper areas (mean depth of ~ 1700 m).

**Table 5:** Statistics of the correlations shown in Fig 6. Percentage of the total basin where each type of correlation (positive, red color in Fig 6 or negative, blue color in Fig 6) is present. Mean depth of the nodes (model locations) where each type of correlation is computed. Mean value of all correlation coefficients (R) for each type of relationships.

		Nodes with positive correlation			Nodes with negative correlation		
Model	rcp	% basin	Mean depth (m)	Mean R	% basin	Mean depth (m)	Mean R
MPI	4.5	12.9	321	0.42	54.7	1658	-0.4
	8.5	12.6	232	0.43	64.1	1737	-0.41
EcEarth	4.5	12.9	190	0.44	56.9	1646	-0.39
	8.5	12.7	392	0.4	62.5	1753	-0.41
	**MEAN**	**12.7**	**283**	**0.42**	**59.6**	**1698**	**-0.4**

Similar analysis with SSS anomalies versus biological variables does not reveal any consistent patterns beyond a general negative correlation but much weaker than with SST (analysis not shown).

**Fig 7:**
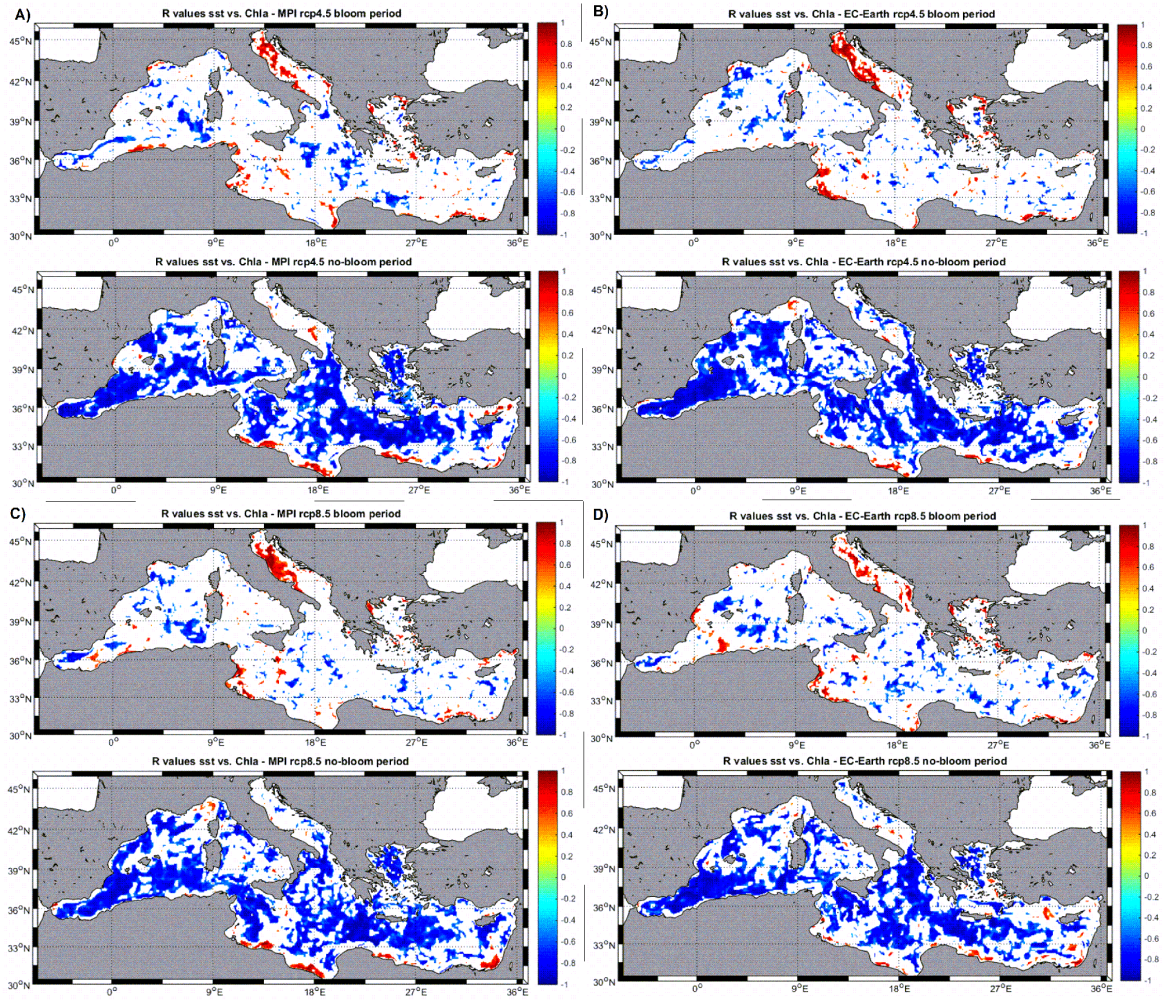
Correlation coefficient (R) maps of monthly SST anomalies vs. Chla anomalies at each model node for the different models/emission scenarios. ‘Bloom’ period corresponds to February-March-April while ‘no-bloom’ period corresponds to July-August-September. Non-significant correlations (at 99%) are blanked.

To understand better the mechanisms behind the relationships shown in Fig 6 and Table 5, a seasonal analysis has been performed in Fig 7. Here, the ‘bloom’ period is defined as the months February, March and April while the ‘non-bloom’ period corresponds to July, August and September according to the typical phytoplankton seasonality in the Mediterranean [31].

For all model forcings there is a clear pattern, during the blooming season the total area of the basin where a significant (either positive or negative) correlation between SST and Chla anomalies is found, is rather small (average ~ 31%, Fig 7) with ~13% showing positive correlations and ~ 18% with negative correlations (Table 6). During the ‘non-bloom’ period there is a much larger fraction of the basin showing significant correlations (average ~ 70%, Fig 7) being the negative correlation much more common (65%) than the positive (5%) (Table 6).

**Table 6:** Statistics of the correlations SST vs. Cla shown in Fig 7. The bloom period corresponds to the months February-April-March while the non-bloom period correspond to the months July-August-September.

		Bloom period (Feb/Apr/March)		No-bloom period (Jul/Aug/Sept)	
Model	rcp	% basin with R>0	% basin with R<0	% basin with R>0	% basin with R<0
MPI	4.5	13.2	18.4	5.5	64.4
	8.5	12.6	18.2	5.1	66.3
EcEarth	4.5	13.9	17.2	4	68.2
	8.5	12.5	21.3	5.5	62.5
	**MEAN**	**13**	**18.7**	**5**	**65.3**

## Discussion

Modifications of freshwater flow into the Mediterranean Sea for the end of the century will not induce large changes in the mean values of the different properties here evaluated (Table 4). All mean changes for hydrological and biogeochemical variables are below 0.4% compared with the baseline simulation; this could be understood considering that the total water flow changes (in annual terms) are between 2.6 km^3^/y and 27 km^3^/y (Table 3), which represent barely between 7.6*10^−5^% and 6.7*10^−4^% of the volume of the Mediterranean basin.

Relative changes in P (and hence in freshwater flow) varies between emission scenarios and between used GCM. As shown in Fig 3, the main differences between MPI and EC-Earth simulated P changes happens in the north-central region of the Mediterranean (i.e., in our *Alpine* and *Adriatic* basins) as for MPI the region will became drier while for EC-Earth it will be wetter. This difference is more acute in RCP4.5 than in RCP8.5 where the different models point to similar P anomalies. It is, however, important to mention that the proposed P and freshwater flow changes are not crucial to the main purpose of this manuscript. We do not aim to create plausible freshwater flow scenarios for the future Mediterranean Sea but only to evaluate how a change in this flow will affect the properties of this marine ecosystem. In this sense, the variety of P changes simulated by the different GCMs is particularly appropriate for our aim as we can test the impacts of a wide range of freshwater flow modifications.

SSS anomalies show clear regional patterns (Fig 4) depending on the specific combination of model/RCP. For example, both realizations using MPI as boundary conditions show a positive SSS anomaly within the Adriatic Sea, stronger under RCP4.5 (Fig 4). This SSS increase is linked with the freshwater flow decrease in the *Alpine* region (Fig 3A and 3C) happening especially during summer and fall for both emission scenarios (Fig 2A and 2C) as described also in previous works [32]. Regarding the EC-Earth forced simulations, only under RCP4.5 coherent SSS anomalies pattern could be seen (Fig 4B) and, surprisingly, the opposite trend is observed, with a fresher Adriatic Sea. This freshening corresponds to the substantial and consistent increase in freshwater flow for the *Alpine* and *Adriatic* regions simulated by this model realization (Fig 3B) with a mean annual increase of ~11%. The substantial increase in precipitation for these regions is consistent with the trend found by [33] and [34] for three different emission scenarios.

SSS changes in other regions of the Mediterranean as, for example, the Balearic Sea under MPI-RCP4.5 are more difficult to interpret. In this particular case, P decreases both in the France and Iberian regions (Fig 3A) so the SSS decrease should not be linked to an increased freshwater flow. Changes in other oceanographic processes such as surface currents and vertical mixing could be responsible for this pattern [35] although a detailed study is beyond the scope of the present contribution. It is also worth mentioning that the SSS anomalies range caused by the freshwater flow modification alone (-0.2–0.8) is of similar magnitude (0.5–0.9) as the one simulated by changing only the atmospheric forcing (*i*.*e*., the baseline simulation) [13].

On the other hand, no clear structures could be detected for both SST and phytoplankton biomass anomalies (Fig 4). Also, the magnitude of induced changes in both variables is sensibly lower than the ones associated to a changing climate in the baseline simulation [13]. It is, however, striking that the effects in terms of these two variables could be detected throughout the entire basin, even in locations very far away from any freshwater source. Indeed, the effects on SST and plankton biomass are much more widespread than salinity changes, so the causing mechanism could not be a simple change of water properties resulting from the subtraction of a certain amount of freshwater flow.

A suitable mechanism that could explain the observed SST anomalies could be the alteration of the vertical water stratification and, hence, of the vertical mixing intensity. The effect of rivers’ outflow on seawater vertical stratification has been described in previous works [36] as it critically influences water renovation rate at the bottom and, hence, oxygen levels. Bottom hypoxia is one of the main symptoms of eutrophication in coastal marine systems [37] so its link with rivers’ discharges has been studied in different marine regions [38]. For the Mediterranean Sea, it has been described that hypoxic conditions in different coastal areas are dependent on rivers conditions, being chemical freshwater quality the most important parameter for some regions while total freshwater flow is more relevant for another areas [39]. However, research on large-scale consequences for marine ecosystems of freshwater flow modifications are absent (to the best of our knowledge) in the literature, making this contribution one of a kind.

Our results indicate that altering the amount of freshwater flow, even if relatively low in absolute terms as described above, could change the baroclinic balance of the whole basin, provoking changes in the vertical mixing intensity throughout the entire Mediterranean Sea. Places with enhanced vertical mixing will present lower SST than in the baseline run and those with decreased mixing will appear warmer. At the same time, more intense mixing will provoke larger fertilization of the upper water column and, hence, increased phytoplankton biomass. This might be the reason of the negative, significant correlation between SST anomalies and phytoplankton biomass anomalies in open water regions (blue regions in Figs 6 and 7). The fact that the negative correlation between SST and Chla is more common during the ‘non-bloom’ season (*i*.*e*., summer, stratified period) indicates that, indeed, the modification of water column vertical stability is a very probably cause for this relationship.

On the contrary, in coastal regions, the water depth is not large enough to allow a successful fertilization of the surface by vertical mixing. Henceforth, the positive correlation between SST anomalies and phytoplankton biomass anomalies in such places could be a mere consequence of the physiological reaction of growth rates to warmer temperatures [40]. The fact that most of this positive correlations are found during the ‘bloom’ period (winter) (Fig 7 and Table 6) seems to support this hypothesis as during this period nutrient supply to the surface waters is typically high so a higher SST could foster higher metabolic rates and plankton growth.

**Fig 8:**
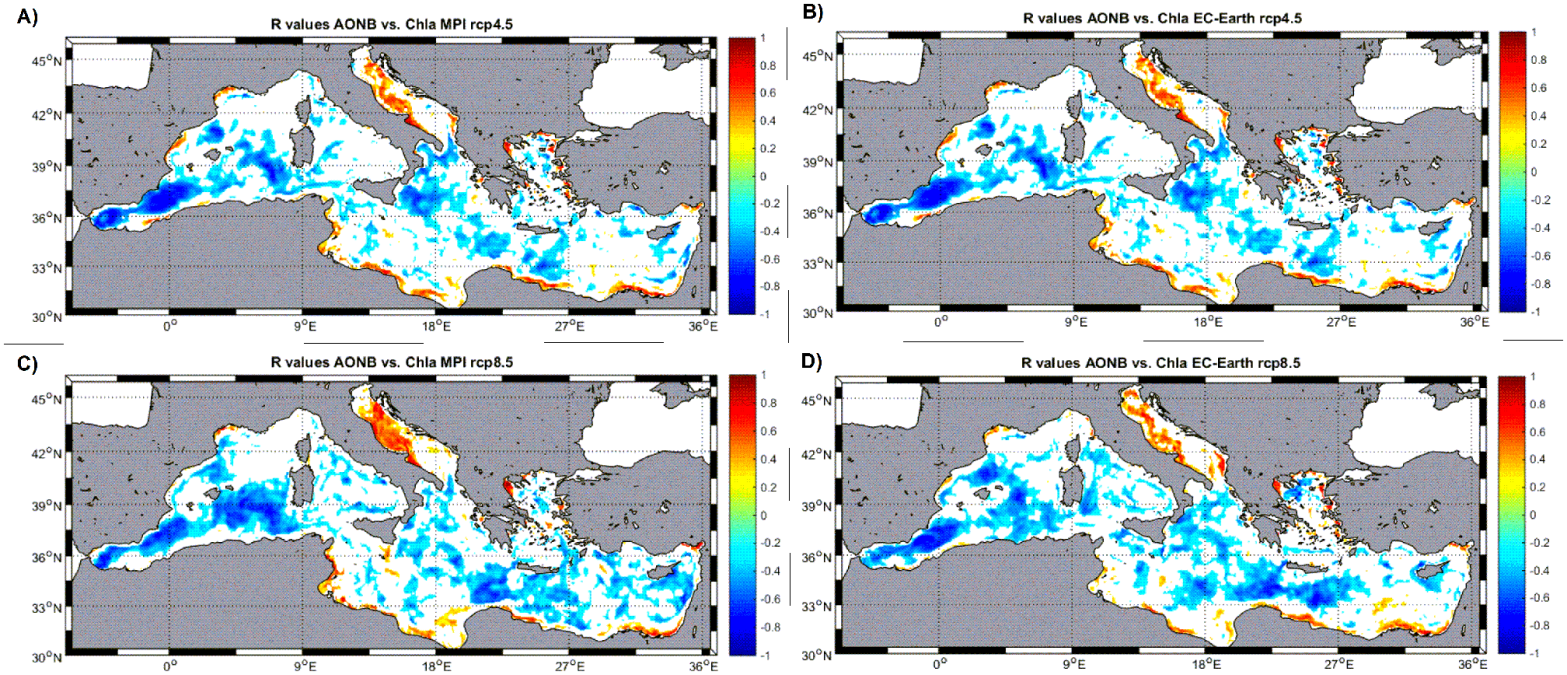
Correlation coefficient (R) maps of monthly AONB (α/β) anomalies vs. Chla anomalies at each model node for the different models/emission scenarios. Non-significant correlations (at 99%) are blanked.

In order to further explore if stratification changes could be responsible for the observed phytoplankton anomalies, the AONB anomalies (see Material and methods) were computed. A negative AONB anomaly indicates a destabilization of the water column while a positive change is related with an increase on vertical stability. The correlation maps of ANOB anomalies versus phytoplankton biomass anomalies (Fig 8) show almost identical patterns as with SST anomalies (Fig 6) with negative coefficients in deep, open water places and positive in coastal regions. This is a further confirmation of the hypothesis presented above: when the water column becomes more unstable (negative AONB anomalies), phytoplankton biomass increases in regions which are deep enough. As stability in shallow regions increases when phytoplankton biomass is larger, this also supports our second hypothesis, that there the increased biomass is likely a result of higher metabolic rates and not caused by higher nutrient supply from deeper layers.

In conclusion, our set of simulations has indicated that a change in the amount of freshwater flow into the Mediterranean Sea, albeit small in comparison with total volume of the basin, could provoke widespread changes in its physical and biogeochemical properties. Salinity changes are more regionally located while temperature and phytoplankton biomass show anomalies in regions quite far away from any freshwater source. The reason behind these last modifications seems to be an alteration of the vertical stratification properties of the water column that changes the vertical mixing intensity and, hence, new surface production triggered by deep nutrients supply.
